# Increased Axin expression enhances adult hippocampal neurogenesis and exerts an antidepressant effect

**DOI:** 10.1038/s41598-018-38103-3

**Published:** 2019-02-04

**Authors:** Wei-Wei Chen, Wing-Yu Fu, Yi-Ting Su, Wei-Qun Fang, Amy K. Y. Fu, Nancy Y. Ip

**Affiliations:** 1 Division of Life Science, The Hong Kong University of Science and Technology, Clear Water Bay, Hong Kong, China; 2 Molecular Neuroscience Center, The Hong Kong University of Science and Technology, Clear Water Bay, Hong Kong, China; 3 State Key Laboratory of Molecular Neuroscience, The Hong Kong University of Science and Technology, Clear Water Bay, Hong Kong, China; 4 Guangdong Provincial Key Laboratory of Brain Science, Disease and Drug Development, HKUST Shenzhen Research Institute, Shenzhen, Guangdong, China

## Abstract

Major depressive disorders are emerging health problems that affect millions of people worldwide. However, treatment options and targets for drug development are limited. Impaired adult hippocampal neurogenesis is emerging as a key contributor to the pathology of major depressive disorders. We previously demonstrated that increasing the expression of the multifunctional scaffold protein Axis inhibition protein (Axin) by administration of the small molecule XAV939 enhances embryonic neurogenesis and affects social interaction behaviors. This prompted us to examine whether increasing Axin protein level can enhance adult hippocampal neurogenesis and thus contribute to mood regulation. Here, we report that stabilizing Axin increases adult hippocampal neurogenesis and exerts an antidepressant effect. Specifically, treating adult mice with XAV939 increased the amplification of adult neural progenitor cells and neuron production in the hippocampus under both normal and chronic stress conditions. Furthermore, XAV939 injection in mice ameliorated depression-like behaviors induced by chronic restraint stress. Thus, our study demonstrates that Axin/XAV939 plays an important role in adult hippocampal neurogenesis and provides a potential therapeutic approach for mood-related disorders.

## Introduction

Major depressive disorder is a critical health problem that affects millions of people worldwide^1^. Impaired adult hippocampal neurogenesis is implicated in the pathogenesis of depression^2^. In contrast, increasing adult hippocampal neurogenesis can buffer stress response and is required for the beneficial effects of several antidepressants^3^. Therefore, the “neurogenic hypothesis” of depression has gained attention because it offers an entry point for identifying molecular targets for therapeutic development. However, the precise molecular mechanisms underlying adult hippocampal neurogenesis and how it regulates the mood are unclear.

Adult neurogenesis in the subgranular zone of the hippocampal dentate gyrus involves multiple well-orchestrated processes initiate upon the activation of neural progenitor cells (NPCs). Within the first week after birth, NPCs activate, proliferate, and subsequently differentiate into intermediate progenitors and neuroblasts. From 2–8 weeks of age, neuroblasts migrate a short distance and gradually mature into granule neurons; during this period, they exhibit enhanced synaptic plasticity, which is thought to be responsible for the unique functions of adult neurogenesis^4^. After 8 weeks of age, the adult-born neurons finally mature and become almost indistinguishable from embryonic-born neurons^5^. Each stage of adult neurogenesis is essential for ensuring proper neuron generation and the maintenance of normal hippocampal function.

The fine-tuned developmental processes of adult neurogenesis are closely coordinated and require the orchestration of multiple intrinsic and extrinsic regulators. Extrinsically, the neurogenic niche, which includes blood vessels, growth factors, endothelial cells, astrocytes, and microglia, triggers signaling cascades in the intracellular compartments to maintain the balance between the proliferation and differentiation of NPCs^6^. Intrinsically, epigenetic regulators, transcription factors, and distinct signaling pathways also nurture the development of NPCs and guide their fates^7–9^. In particular, deficits in pathways such as Notch, Hedgehog (Shh), bone morphogenetic protein (BMP), and Wnt signaling lead to impaired adult neurogenesis and are closely associated with the development of mood and psychiatric disorders such as anxiety, major depression, and cognitive impairment^10,11^. Therefore, studies of the molecular and cellular mechanisms underlying adult neurogenesis will advance our understanding of the association between adult neurogenesis and psychiatric disorders.

Axis inhibition protein (Axin) is a scaffold protein that was originally identified to inhibit axis formation during development^12^. Through its association with a plethora of signaling pathways such as the Wnt, Notch, and BMP pathways, Axin is also involved in guiding neuronal migration, mediating axon formation, and regulating synaptic morphogenesis during nervous system development^13–15^. We previously demonstrated that Axin is expressed in embryonic NPCs during cerebral development and that its subcellular localization regulates the amplification and differentiation of NPCs^16^. Furthermore, an aberrant increase of Axin in the cerebral cortex during development leads to the overexpression of upper-layer neurons; this results in an imbalance between excitatory and inhibitory neurotransmission, which is strongly associated with the development of psychiatric disorders such as social deficits and autism^17^. Given that an elevated Axin level enhances neurogenesis during embryonic brain development, we hypothesized that Axin enhances adult hippocampal neurogenesis and/or adult neurogenesis-related brain functions.

Accordingly, in the present study, we showed that increasing Axin protein level with XAV939, a small molecule Axin stabilizer^18^, robustly promoted adult neurogenesis, rescued stress-induced impairment of adult neurogenesis, and alleviated anxiety/depression behaviors under stressful conditions. Specifically, stabilization of Axin by XAV939 facilitated the proliferation of adult NPCs and neuron production in the adult mouse hippocampus. Importantly, this enhancement of adult neurogenesis ameliorated depression-like behaviors in mice, such as anhedonia and learned helplessness under chronic stress environments. Therefore, our study provides experimental evidence indicating that administration of small molecules targeting Axin, such as XAV939, can enhance adult hippocampal neurogenesis and regulate mood modulation. Thus, Axin is a potential molecular target for drug development for major depressive disorders.

## Results

### Elevated Axin protein level enhances adult NPC proliferation and neuron production

**Figure 1:**
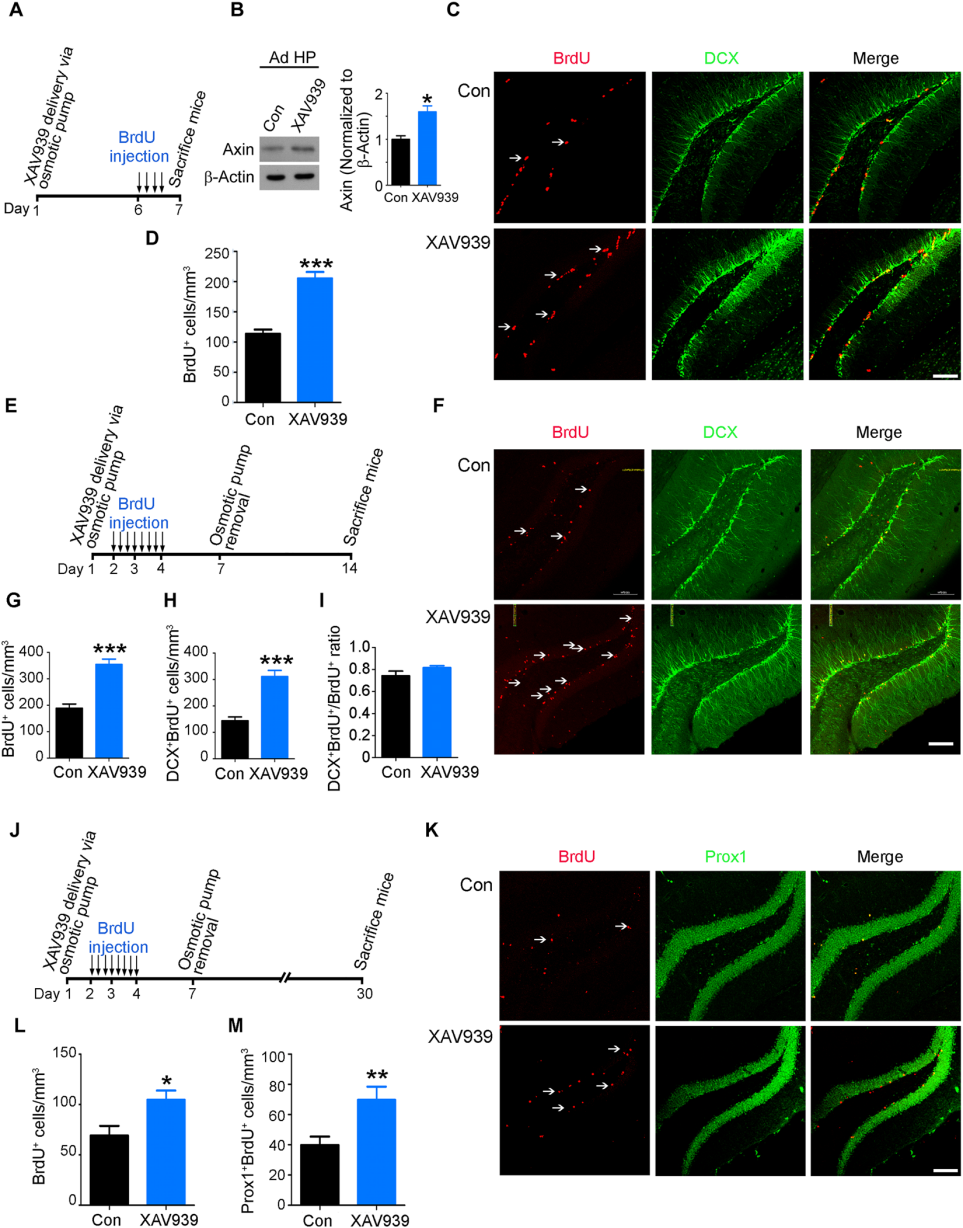
XAV939, an Axin stabilizer, enhances the proliferation of neural progenitor cells (NPCs) and neuron production in the adult mouse hippocampus. (**A**–**D**) XAV939 administration promoted the proliferation of adult hippocampal NPCs. (**A**) XAV939 and BrdU administration paradigm. (**B**) XAV939 administration increased Axin protein level in the adult mouse hippocampus. Western blots of Axin protein (actin as a loading control; left panels) and quantification graph (right panel); *n* = 3 mice per group. (**C**,**D**) XAV939 administration for 7 days increased the newly formed proliferating NPCs (BrdU^+^ cells; *n* = 4 mice per group). Representative images of the dentate gyrus (**C**) and quantification graph (**D**). (**E**–**I**) XAV939 enhanced the survival of NPCs and neuron production in the adult hippocampus. (**E**) XAV939 and BrdU administration paradigm. Representative images of the dentate gyrus (**F**) and quantification graphs showing the number of NPCs (BrdU^+^ cells); (**G**) newly differentiated neurons (DCX^+^BrdU^+^ cells); (**H**) and the ratio of newborn neurons (DCX^+^BrdU^+^) versus BrdU^+^ cells (DCX^+^BrdU^+^ cells/BrdU^+^); (**I**) 14 days after XAV939 administration (*n = *4 mice per group). (**J**–**M**) XAV939 administration enhanced the integration of newly differentiated neurons into the dentate gyrus in the adult hippocampus. (**J**) Schedule of XAV939 and BrdU administration. Representative images of the dentate gyrus (**K**) and quantification graphs showing the BrdU^+^ cells (**L**) and newly formed neurons expressing Prox1 (Prox1^+^BrdU^+^ cells; (**M**) 30 days after XAV939 administration (*n* = 3 mice per group). The white arrows indicate the BrdU^+^ cells in the dentate gyrus. *p < 0.05, **p < 0.01, ***p < 0.001, Student’s *t*-test. Scale bar = 100 μm.

To investigate whether Axin is involved in adult hippocampal neurogenesis, we first examined its cellular localization in the dentate gyrus of the adult mouse hippocampus. We found that Axin is widely expressed in different neural cell types including NPCs, neuroblasts, and mature neurons in the dentate gyrus regions (Supplementary Figure S1A). In particular, we observed Axin protein in dividing NPCs labeled with 5-bromo-2′-deoxyuridine (BrdU) in the subgranular zone of the dentate gyrus in the adult mouse brain (Supplementary Figure S1A). As this finding suggests that Axin might regulate adult neurogenesis, we increased the Axin protein level in the mouse brain by administering XAV939, a tankyrase inhibitor that specifically stabilizes Axin protein^18^. XAV939 successfully increased the Axin protein level in adult NPC cultures (Supplementary Figure S1B). To examine the effects of increasing Axin at different stages of adult hippocampal neurogenesis, i.e., NPC proliferation, survival, and maturation^9^, we injected BrdU into mice at different time points during or after XAV939 treatment (Fig. 1A,E,J). First, we examined whether increasing Axin level regulates NPC proliferation by labeling and tracking the proliferation of newly dividing NPCs with BrdU 6 days after XAV939 administration (Fig. 1A,C,D). Compared to the control mice, XAV939 administration markedly increased Axin protein level in the adult hippocampus (Fig. 1B). Moreover, increased Axin expression led to increased β-cateinin phosphorylation and resulted in the reduced expression of β-catenin (Supplementary Figure S1C,D), suggesting that XAV939 administration inhibits Wnt/β-catenin signaling through Axin stabilization *in vivo*. Importantly, compared to the controls, XAV939-treated mice had significantly more BrdU^+^ cells (Fig. 1C,D) and Ki67^+^ proliferating cells (Supplementary Figure S1E,F) in the subgranular zone, indicating that increased Axin protein level enhances NPC proliferation in the dentate gyrus. Moreover, XAV939 did not affect cell death in the XAV939-treated mice, as there was no induction of apoptotic marker cleaved-caspase-3 staining (Supplementary Figure S1G). Next, we determined if increased Axin level regulates the survival or differentiation of NPCs. On the second day after XAV939 administration, we labeled the newly divided NPCs by injecting BrdU for 2 days and tracked their fate (i.e., survival and neuronal differentiation) for approximately 2 weeks (Fig. 1E). Compared to the controls, the XAV939-treated mice exhibited significantly more BrdU^+^ cells in the subgranular zone (Fig. 1F,G), indicating that increased Axin level can maintain the survival of the newly generated NPCs. In addition, XAV939-treated mice exhibited an increase of DCX^+^BrdU^+^ immature neurons, although the proportion of DCX^+^BrdU^+^ neurons among BrdU^+^ cells remained relatively unchanged compared to the control mice (Fig. 1F,H,I). These results collectively demonstrate that increasing Axin protein level supports the proliferation of NPCs and maintains their survival, which subsequently leads to increased production of immature neurons.

Next, to examine whether the immature neurons induced by XAV939 can mature into granule neurons and integrate into the dentate gyrus circuitry, we sacrificed the mice approximately 3 weeks after XAV939 and BrdU administration (Fig. 1J) and stained hippocampal sections with a mature neuron marker, prospero-related homeobox 1 (Prox1). Compared to the control mice, XAV939-treated mice had more BrdU^+^ cells (Fig. 1K,L) and newly generated granule neurons (Prox1^+^ BrdU^+^) in the hippocampus (Fig. 1K,M). This suggests that XAV939 treatment enhances the production of immature neurons, which ultimately differentiate into granule neurons. Therefore, these results collectively suggest that XAV939 promotes the amplification of adult NPCs and neuron production in the mouse dentate gyrus without affecting the survival of NPCs.

### XAV939 alleviates depression-like behavior

**Figure 2:**
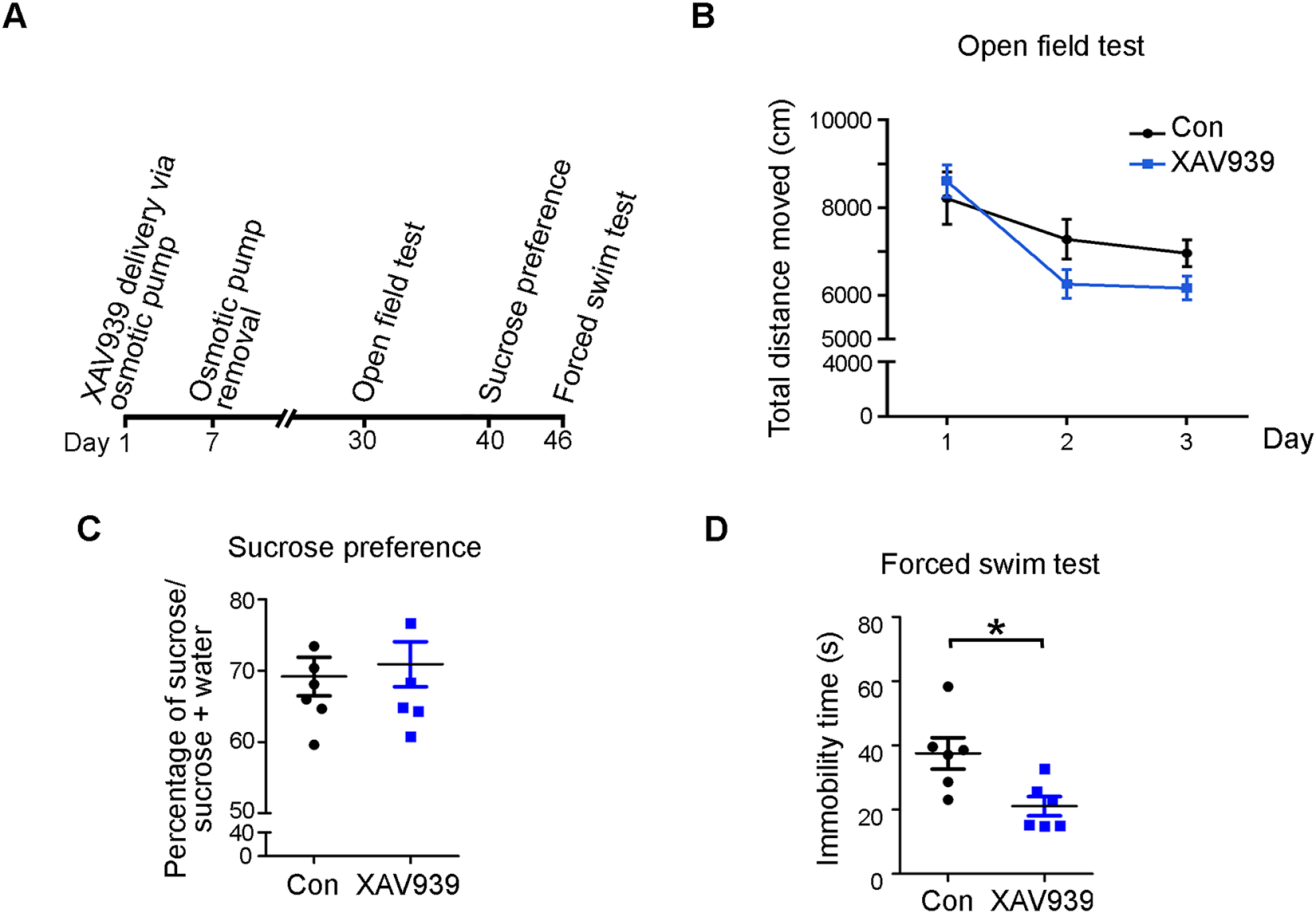
XAV939 exerts an antidepressant effect. (**A**) Timeline of XAV939 administration and depression-related behavioral tests. (**B**) XAV939 did not affect motor activity in mice in the exploratory open field test. Graph shows how far the mice travelled each day for 3 consecutive days. (**C**) XAV939 administration did not change sucrose consumption in the sucrose preference test. (**D**) XAV939 treatment reduced the immobility time of mice in the forced swim test (*n* = 6–7 mice per group, *p < 0.05, Student’s *t*-test).

Given that adult hippocampal neurogenesis is associated with emotional behaviors^19^, it is of interest to determine whether Axin-dependent adult hippocampal neurogenesis regulates the affective status of mice. Accordingly, we assessed the emotional behaviors of mice 30 days after XAV939 administration, when the newly generated neurons were integrated into the neural circuitry (Fig. 2A). Compared to the control mice, the XAV939-treated mice did not exhibit any obvious difference in motor or exploratory activity in the open field test (Fig. 2B). The XAV939-treated mice behaved similar to the control mice in the sucrose preference test (Fig. 2C), suggesting that XAV939 does not affect the emotional behaviors of anhedonia under normal conditions. In addition, compared to the control mice, XAV939-treated mice exhibited significantly less immobility in the forced swim test (Fig. 2D), a behavioral model of learned helplessness widely used to assess depressive behavior. Collectively, these results suggest that XAV939 treatment ameliorates depression-like behaviors in mice.

### XAV939 rescues the chronic restraint stress-induced impairment of adult hippocampal neurogenesis

Stress-induced depression is strongly associated with impaired adult hippocampal neurogenesis. Therefore, we further examined the beneficial antidepressant-like effect of XAV939 in mice subjected to chronic restraint stress (CRS), which is a well-established animal model of inducing depression-like behaviors and is associated with the impairment of adult hippocampal neurogenesis^20^. On days 11 and 21 after stress induction, mice exhibited a significantly elevated plasma level of corticosterone (Supplementary Figure S2), which served as an indicator of depression^21^. Compared to the unstressed mice, the mice subjected to CRS showed a slight decreasing trend in locomotor activity in the open field test (Supplementary Figure S3A,B). However, the CRS mice elicited robust hyponeophagia-like behavior as demonstrated by increased latency to approach and eat the food pellet in the novelty-suppressed feeding test (Supplementary Figure S3C). In addition, compared to the unstressed mice, the CRS mice exhibited anhedonia and despair as demonstrated by decreased sucrose consumption in the sucrose preference test (Supplementary Figure S3D) and increased immobile time in the forced swim test (Supplementary Figure S3E), respectively. These results validate the use of CRS as an animal model of depression to examine the functional consequence of XAV939 treatment under stressful conditions.

**Figure 3:**
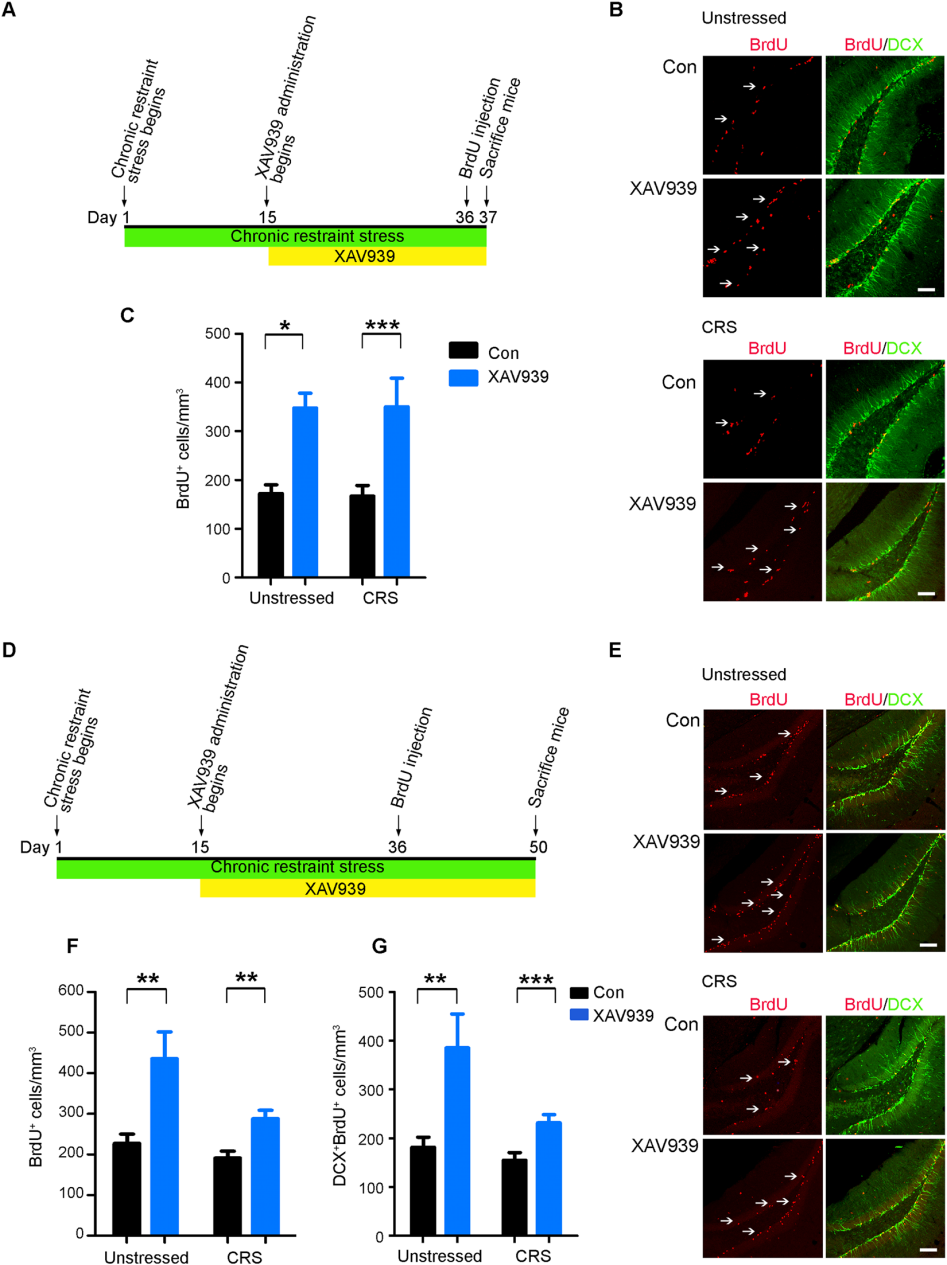
XAV939 induces hippocampal neurogenesis in adult mice under chronic restraint stress. (**A**–**C**) XAV939 increased the NPCs proliferation in the dentate gyrus under chronic restraint stress (CRS). (**A**) Timeline of XAV939 administration, BrdU injection, and CRS induction. (**B**) Representative images of BrdU and DCX staining in the hippocampus of mice treated with vehicle (Con) or XAV939 under free-moving (unstressed) or CRS conditions. (**C**) Quantification showing the proliferation of neural progenitor cells (NPCs; BrdU^+^ cells; *n* = 14 slices from 3 mice per group, *p < 0.05, ***p < 0.001, two-way ANOVA with Bonferroni *post hoc* test). (**D**–**G**) XAV939 enhanced the generation of NPCs and neuron production in the dentate gyrus under CRS. (**D**) Timeline of XAV939 administration, BrdU injection, and CRS induction. (**E**) Representative images of BrdU and DCX staining in the hippocampus of mice treated with vehicle (Con) or XAV939 under unstressed or CRS conditions. Quantification showing the number of NPCs (BrdU^+^ cells); (**F**) and the differentiation of NPCs into immature neurons (DCX^+^BrdU^+^ cells); (**G**) (*n* = 14 slices from 3 mice per group, **p < 0.01, ***p < 0.001, two-way ANOVA with Bonferroni *post hoc* test). The white arrows indicate the BrdU^+^ cells in the dentate gyrus. Scale bar = 100 μm. Unstressed: mice allowed to move freely during the course of the experiment; CRS: chronic restraint stress-induced mice.

Accordingly, we used BrdU labeling and cell fate tracking to determine if XAV939 rescues the CRS-induced impairment of adult hippocampal neurogenesis. XAV939 treatment increased the number of BrdU-labeled NPCs in the subgranular zone in CRS mice (Fig. 3A–C). To track the survival and generation of the newborn NPCs under CRS insult, we subjected the mice to CRS and XAV939 administration for an additional 2 weeks after BrdU labeling (Fig. 3D). We found that XAV939 significantly increased the number of BrdU^+^ cells in CRS mice, although not to the same extent as that in unstressed mice (Fig. 3E,F). This suggests that XAV939 administration also exerts a protective effect on the newly dividing NPCs under stressful conditions. Furthermore, XAV939 increased the number of immature neurons in CRS mice (Fig. 3E,G), suggesting that XAV939 can rescue the CRS-mediated impaired production of immature neurons. Hence, these results collectively suggest that XAV939 rescues the proliferation, survival, and differentiation ability of adult NPCs in mice subjected to CRS.

### XAV939 rescues depression-like behaviors in CRS mice

**Figure 4:**
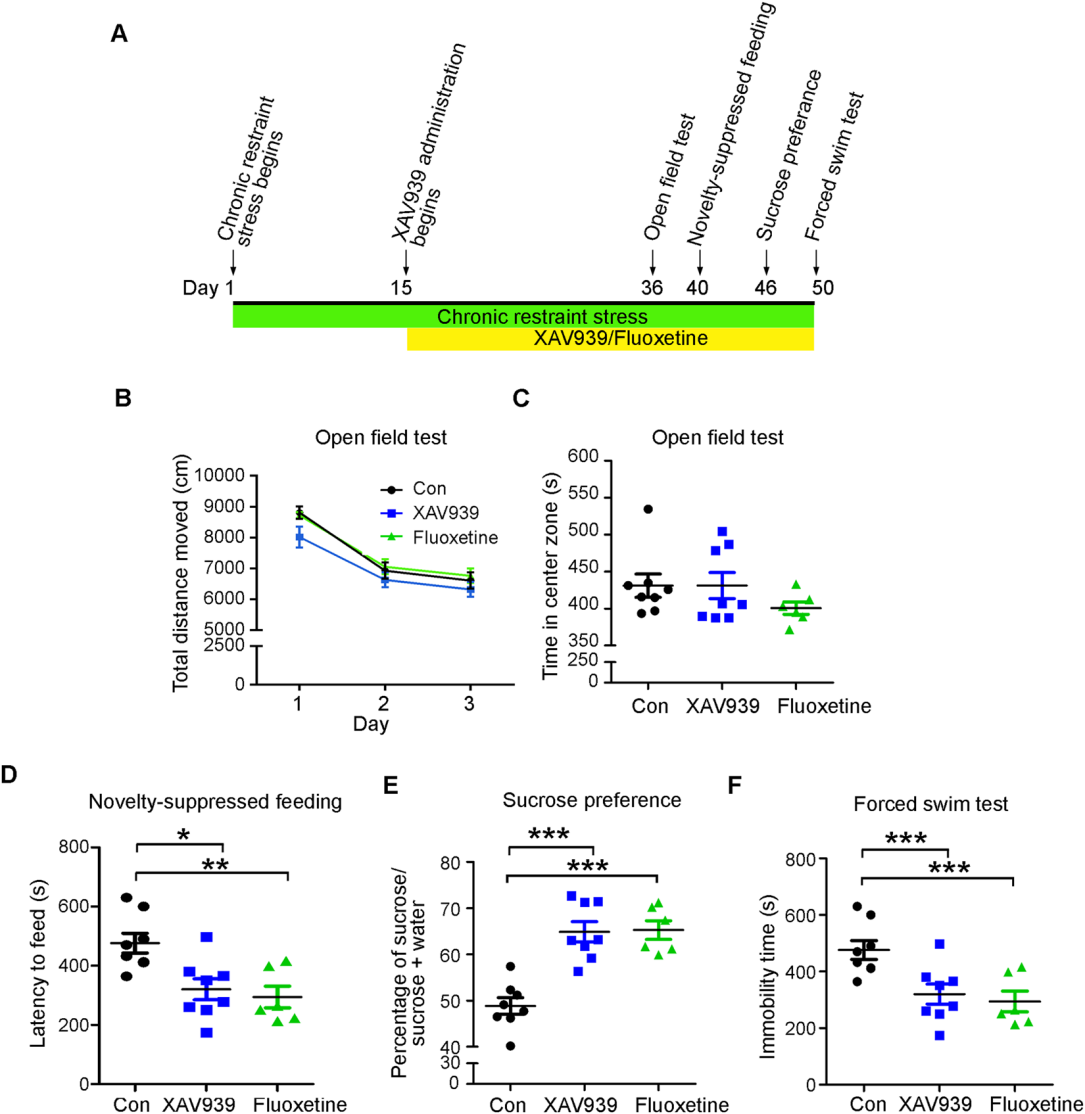
XAV939 rescues chronic restraint stress-induced depression-like behaviors. (**A**) Timeline of XAV939 administration, chronic restraint stress (CRS) induction, and depression-related behavioral tests. Fluoxetine was used as a positive control. (**B**,**C**) XAV939 did not affect the motor activity or anxiety of CRS mice in the open field test. (**B**) Motor activity of the CRS mice treated with XAV939 or fluoxetine in the 3-day open field test. (**C**) XAV939 treatment did not affect the time CRS mice spent in the center zone of a novel arena in the first day of the open field test. (**D**) XAV939-treated CRS mice exhibited reduced latency to feed in the novelty-suppressed feeding test. (**E**) XAV939-treated CRS mice exhibited increased sucrose consumption in the sucrose preference test. (**F**) XAV939-treated CRS mice exhibited reduced immobility time in the forced swim test (*n* = 6–8 mice per group; *p < 0.05, **p < 0.01, ***p < 0.001, one-way ANOVA).

Given that XAV939 enhanced the proliferation and neuronal generation of adult NPCs under stressful conditions, we further examined whether XAV939 administration ameliorates the CRS-induced depression-like behaviors in mice (Fig. 4A). We found that in mice subjected to CRS, XAV939 administration did not affect their motor activity or exploratory behaviors in the open field test (Fig. 4B,C). Notably, compared to the control mice, the XAV939-treated stressed mice exhibited significantly reduced latency to feed in the novelty-suppressed feeding test (Fig. 4D) as well as increased sucrose consumption in the sucrose preference test (Fig. 4E). In addition, compared to the control mice, the XAV939-treated stressed mice exhibited reduced immobility in the forced swim test (Fig. 4F), indicating that XAV939 ameliorates the depression-like behaviors in this learned helplessness model. Thus, these results collectively suggest that XAV939 administration enhances adult neurogenesis and buffers stress-induced depression-like behaviors.

## Discussion

In the present study, we found that stabilizing Axin protein by administering the small molecule XAV939 enhanced adult hippocampal neurogenesis and ameliorated stress-induced affective disorders. Specifically, in addition to increasing Axin expression, XAV939 administration enhanced the proliferation and survival of NPCs, which subsequently enhanced the generation of mature neurons that integrated into the adult hippocampal circuitry. Thus, our findings collectively demonstrate that increasing Axin level uniquely contributes to enhancing adult hippocampal neurogenesis and ameliorating depression-like behaviors. Therefore, stabilizing Axin protein is a potential stem cell-based therapeutic approach to replace neurons lost because of neuronal degeneration. Moreover, XAV939 might be a good candidate for drug development for adult neurogenesis-associated depressive disorders.

What are the mechanisms that underlie the beneficial effect of XAV939 on adult hippocampal neurogenesis? Given that XAV939 is a tankyrase inhibitor that stabilizes Axin expression in the canonical Wnt pathway, the molecular nature of Axin can be scrutinized in this context. Axin is a scaffold protein whose multiple functional roles are determined by its interactions with various signaling proteins through multiple domains. Canonically, Axin mediates the phosphorylation and degradation of β-catenin in the absence of Wnt ligand^15,22^. Because β-catenin is a critical transcriptional factor that antagonizes NPC amplification and promotes neuronal differentiation^23^, XAV939 might stabilize Axin to promote NPC proliferation by negatively regulating the canonical Wnt/β-catenin pathways in the adult hippocampus (Supplementary Figure S1C, D). In addition, given that Axin independently interacts with other pathways that actively regulate adult hippocampal neurogenesis, such as the Notch/RBPJ, BMP, and TGFβ signaling pathways^24–26^, the actions of XAV939 might involve other signaling pathways at distinct stages of adult neurogenesis via Axin regulation.

The beneficial effect of XAV939 on depression-like behaviors in stressed mice raises the question of how XAV939 endows this antidepressant-like response. Our finding that XAV939 administration can increase adult neurogenesis under stressed conditions without affecting basal anxiety or depression level is consistent with the literature, i.e., adult neurogenesis has a unique role in helping animals cope specifically with stress not under basal conditions^3,19,27^. XAV939 likely promotes adult neurogenesis in the dentate gyrus, and the resultant increase of newborn neurons consequently provides resilience to CRS, ameliorating anxiety/depression-like behaviors. In addition, because the dentate gyrus is synaptically connected to the hypothalamus and amygdala, which actively regulate affective states^19^, XAV939-stimulated adult neurogenesis might facilitate the neural circuitry connections between these regions to mediate mood control. Specifically, CRS induces the release of the stress hormone corticosterone from the hypothalamic–pituitary–adrenal axis, which negatively reduces adult neurogenesis^28–30^. Given that XAV939 can rescue the CRS-mediated impairment of neurogenesis (Fig. 3), it is of interest to further resolve the interaction between XAV939 and the hypothalamic–pituitary–adrenal axis in order to determine whether XAV939 directly or indirectly interacts with this axis or stress hormones to help animals cope with chronic stress.

XAV939 was first discovered to specifically inhibit Wnt signaling via its tankyrase 1 (TNKS1) and tankyrase 2 (TNKS2) activities towards Axin^18^. Given the high similarity between TNKS1/2 and other poly (ADP-ribose) polymerase (PARP) family members, XAV939 might also inhibit other PARP family members (e.g., PARP1 and PARP2), albeit to a lesser extent^18^. Of note, patients with major depressive disorders have elevated PARP1 levels^31^. Moreover, in a recent study, PARP inhibitors exhibited antidepressant effects in the sucrose preference and forced swim tests, similar to the effects of fluoxetine^32^. Hence, further investigation is warranted to determine whether XAV939 exerts its antidepressant effects through the modulation of PARP activity.

In summary, the findings of the present study provide insights into the roles of Axin in enhancing adult hippocampal neurogenesis under basal or chronic stress conditions. Thus, elucidating the beneficial effects of XAV939/Axin signaling in adult hippocampal neurogenesis and depressive behaviors will contribute to the development of effective therapies for psychiatric disorders.

## Methods

### Animals

Mice were bred in the Animal and Plant Care Facility of The Hong Kong University of Science and Technology and handled in accordance with the Animals (Control of Experiments) Ordinance of Hong Kong. All animal experiments were performed in accordance with protocol #2017064, which was approved by the Animal Care Committee of The Hong Kong University of Science and Technology.

### Chemicals and antibodies

XAV939 was purchased from Tocris Bioscience. BrdU and fluoxetine were from Sigma-Aldrich. The following antibodies were used for immunostaining: anti-Axin (A0481, Sigma-Aldrich), anti-Nestin (MAB353, Millipore), anti-Prox1 (sc-8025, Santa Cruz Biotechnology), anti-NeuN (MAB377B, Millipore), anti-doublecortin (DCX, AB2253; Millipore), anti-BrdU (OBT0030G, AbD Serotec), and anti-GFP (clone 3E6) Alexa Fluor 488-, 546-, and 647-conjugated secondary antibodies (Thermo Fisher).

### Drug delivery

We implanted 10–12-week-old male C57 mice with Alzet mini-osmotic pumps (model 1004), pumping their contents at 0.5 µL/h for 7 days. We loaded the pumps with XAV939 (1 mM), fluoxetine (18 mg·kg^−1^·day^−1^), or vehicle solvent (3% DMSO + 0.2% Tween 20) in PBS (pH 7.4). We adjusted the mini-osmotic pumps intracerebroventricularly in the right hemisphere as previously described^33^. After osmotic pump implantation, we housed the mice individually and collected them for experiments after different periods according to the experimental paradigm in use. We dissolved BrdU in sterilized phosphate-buffered saline (PBS) and delivered it into the mice via intraperitoneal injection (50 mg/kg). The schedules of XAV939 delivery and BrdU administration are illustrated in Fig. 1A,E and J as well as Fig. 3A and D.

### Immunohistochemical analysis

We sacrificed the animals by cardiac perfusion with 4% paraformaldehyde. After perfusion, we quickly removed their brains, post-fixed them in 4% paraformaldehyde at 4 °C for 24–48 h, and sectioned them with a vibratome (Leica). We sectioned the mouse brains from the control and treatment group at 50 μm, spanning the entire dentate gyrus, and maintained the sections in serial order. We used 1 in every 4 brain sections with the same serial number for a total of 5 slices from each mouse for staining and quantification. After permeabilization and blocking in PBS containing 0.4% Triton X-100 and 3% goat serum at room temperature for 1 h, we incubated the mouse brain sections with specific primary antibodies at 4 °C for 36–48 h, followed by corresponding Alexa Fluor-conjugated secondary antibodies at room temperature for 2 h. To label BrdU, we fixed the mouse brain sections with 4% paraformaldehyde at room temperature for 1 h, followed by DNA denaturation with 1 M HCl at 45 °C for 30 min. We subsequently permeabilized the brain slices and blocked them in PBS containing 0.4% Triton X-100 and 3% goat serum at room temperature for 1 h, followed by primary BrdU antibody incubation overnight at 4 °C. After secondary antibody labeling, we mounted the brain sections in hydro-mount medium (HS-106, National Diagnostics).

### Image acquisition and quantitative analysis

We acquired images with an Olympus FluoView FV1000 confocal microscope (Olympus). We collected serial images of brain sections (50 μm thickness) using the z-serial scanning mode (2-μm step with 8–9 optical sections). We quantified BrdU^+^, DCX^+^BrdU^+^ or Prox1^+^BrdU^+^ cells from images at equivalent positions (i.e., rostrocaudal and lateromedial) using MetaMorph software version 7.8.8.0 (Universal Imaging Corporation). We calculated the volume of the dentate gyrus granular layer using ImageJ software. Accordingly, we calculated cell density by dividing the number of BrdU^+^ or DCX^+^BrdU^+^ or Prox1^+^BrdU^+^ cells by the dentate gyrus granular layer volume.

### CRS

We carried out all behavioral tests on male XAV939-injected and unstressed mice at 10–12 weeks of age. The order of the behavioral tests is described in Figs 2A and 4A. Mice were allowed to adapt to a reversed 12-h:12-h light/dark cycle for 1 week before behavioral experiments. We induced CRS by placing each mouse horizontally in a 50-mL conical centrifuge tube (2.7 cm in diameter, 7 cm long) with multiple perforated holes (~2 mm in diameter) that allowed for adequate ventilation^34^. The dimensions of the tube effectively restrained the mice by preventing them from turning around or moving forward or backward. We exposed the mice to CRS for 7.5 hours per day (from 9:30 h to 17:00 h daily) according to the experimental schedule. Meanwhile, the unstressed mice were allowed to move freely in their home cage with food and water *ad libitum*. We measured corticosterone level by enzyme-linked immunosorbent assay (ELISA). The schedules for inducing CRS, XAV939 delivery, and BrdU administration are illustrated in Figs 3A,D and 4A.

### Open field test

We recorded the locomotor activity of mice in the open field test as previously described^35^. In brief, we placed experimental mice in the center of an open-top chamber (50 × 50 × 40-cm). The mice were allowed to explore the chamber for 15 min each day for 3 consecutive days. We recorded locomotor activity in the 3 trials by using video tracking software from Noldus (EthoVision XT) and subsequently determined the distance moved. We cleaned the chamber with 70% ethanol after each trial.

### Novelty-suppressed feeding test

Prior to the novelty-suppressed feeding test, we deprived the mice of food for 24 h and then placed them in a brightly lit open arena for 10 min. A weighed food pellet was placed on a white filter paper at the center of the open arena. We videotaped the behaviors of each mouse and recorded the time it took to start feeding. Upon returning to their home cage, we analyzed the total amount of food consumed during 5 min to test whether feeding differences in the novel environment were due to differences in hunger.

### Sucrose preference test

Prior to the sucrose preference test^17^, the mice were housed individually and supplied with 2 water bottles for 3 days: one containing water, and the other containing 2% sucrose solution. On days 4–6, the mice were free to drink either the liquid. We measured the amounts of water and sucrose solution consumed by each mouse and switched the positions of the two bottles daily. We calculated sucrose preference as follows:

$$({\rm{\Delta }}{\rm{weight}}\,{\rm{sucrose}})\div({\rm{\Delta }}\mathrm{weight}\,{\rm{sucrose}}+{\rm{\Delta }}\mathrm{weight}\,{\rm{water}})\times 100$$

### Forced swim test

For the forced swim test^36^, we placed a mouse into a clear Pyrex cylinder (diameter: 10 cm) filled with tap water at ambient temperature for 10 min of training. On the second day, testing was performed again by placing the mouse in the water-filled cylinder for 6 min. We recorded the whole session on video. We measured the total immobile time of each mouse as indicated by a static vertical posture of the mouse in the last 4 min.

### Statistical analysis

All data are expressed as the arithmetic mean ± standard error of the mean (SEM). The significance of differences was analyzed by Student’s *t-*test, one-way ANOVA, or two-way ANOVA, where appropriate, using GraphPad prism version 6 (GraphPad Software). The level of statistical significance was set at *p < 0.05, **p < 0.01, or ***p < 0.001.

## Supplementary information

Supplementary Info
