# Supplementary material for: Increased Axin expression enhances adult hippocampal neurogenesis and exerts an antidepressant effect
Source: Sci Rep. 2019 Feb 4;9:1190. doi: 10.1038/s41598-018-38103-3 (PMC6362220; doi:10.1038/s41598-018-38103-3)
Supplement: Supplementary file 1 — Supplementary Info [file 41598_2018_38103_MOESM1_ESM.pdf]

SUPPLEMENTARY MATERIAL

Increased Axin expression enhances adult hippocampal neurogenesis and exerts an antidepressant effect

Wei-Wei Chen[1,2,3,a], Wing-Yu Fu[1,2,3,a], Yi-Ting Su[1,2,3], Wei-Qun Fang[1,2,3], Amy K.Y. Fu [1,2,3,4], and Nancy Y. Ip[1,2,3,4*]

[1] Division of Life Science, [2] Molecular Neuroscience Center, [3] State Key Laboratory of Molecular Neuroscience, The Hong Kong University of Science and Technology, Clear Water Bay, Hong Kong, China

[4] Guangdong Provincial Key Laboratory of Brain Science, Disease and Drug Development, HKUST Shenzhen Research Institute, Shenzhen, Guangdong, China

[a] Both authors contributed equally to this work

* Correspondence and requests for materials should be addressed to N.Y.I. (email: boip@ust.hk)

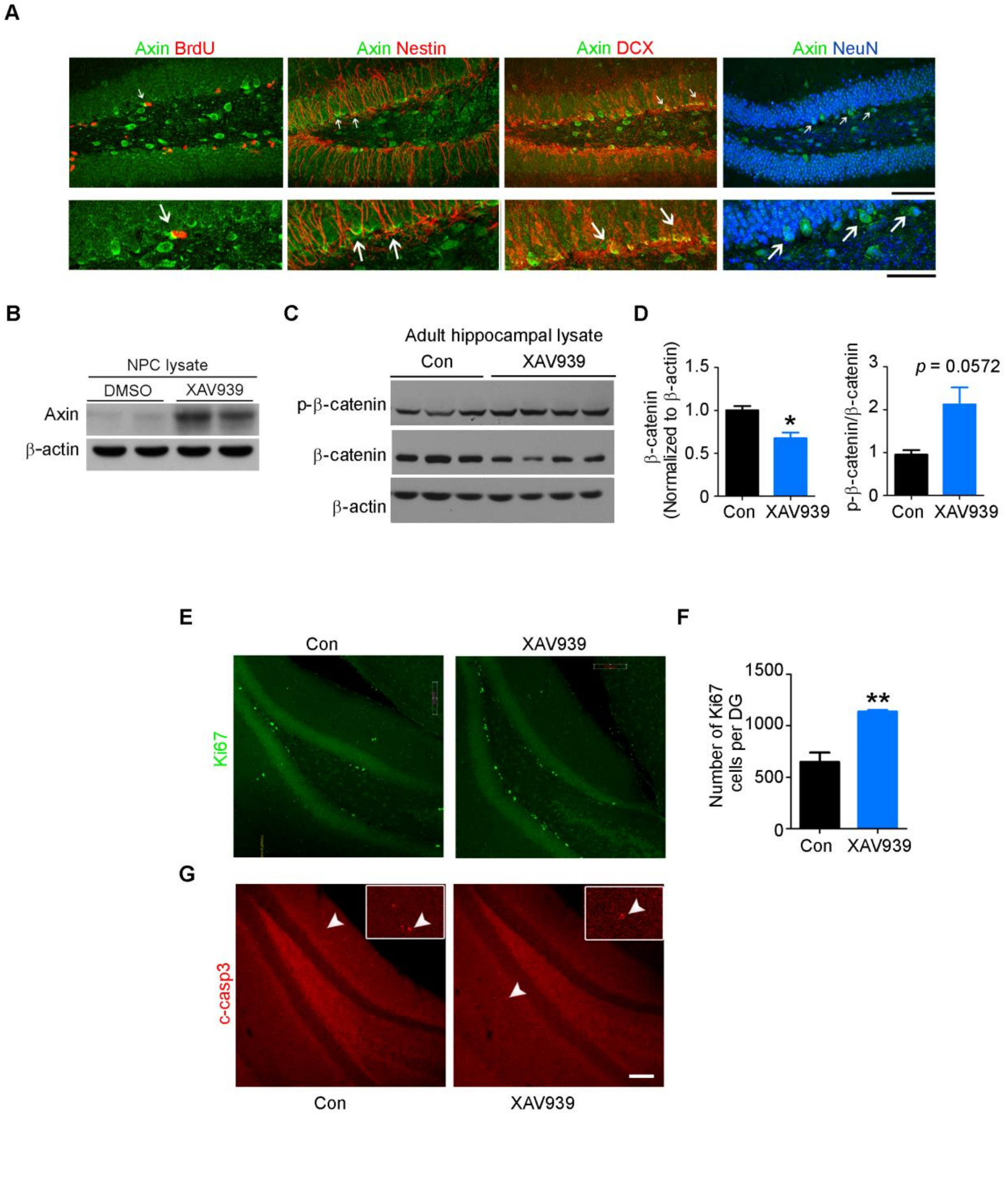

Supplementary Figure S1. Axin protein is expressed in the dividing neural progenitor cells (NPCs) in the dentate gyrus in the hippocampus. (A) Axin was co-localized with BrdU$^+$

proliferating NPCs, Nestin$^+$ NPCs, DCX$^+$ neuroblasts, and NeuN$^+$ neurons in the dentate gyrus of adult mice (arrowheads). Three-month-old mice were injected with BrdU to label dividing cells, and hippocampal slices were subsequently immunostained for Axin, BrdU, Nestin, DCX, and NeuN. Upper panels, scale bar = 50 μm; lower panels, scale bar = 20 μm. (B) XAV939 treatment increased Axin level *in vitro*. Western blot analysis showing Axin protein expression level in adult neural stem cell (NPC) cultures derived from the subgranular zone treated with DMSO control or 5 μM XAV939. (C, D) XAV939 administration regulated the Wnt/β-catenin signaling pathway *in vivo*. Western blots (C) and quantification graph (D) showing the level of phosphorylated β-catenin (p-β-catenin) and total β-catenin level in the adult mouse hippocampus 7 days after XAV939 administration. (E, F) XAV939 administration increased Ki67$^+$ dividing cells in the adult subgranular zone. Representative images (E) and quantification graph (F) showing the increased number of Ki67$^+$ cells 7 days after XAV939 treatment. (G) XAV939 administration did not increase apoptosis in the adult mouse dentate gyrus as indicated by cleaved caspase-3 (c-casp3) immunostaining. Inserts: enlarged representative images showing the c-casp3$^+$ cells. Scale bar = 100 μm.

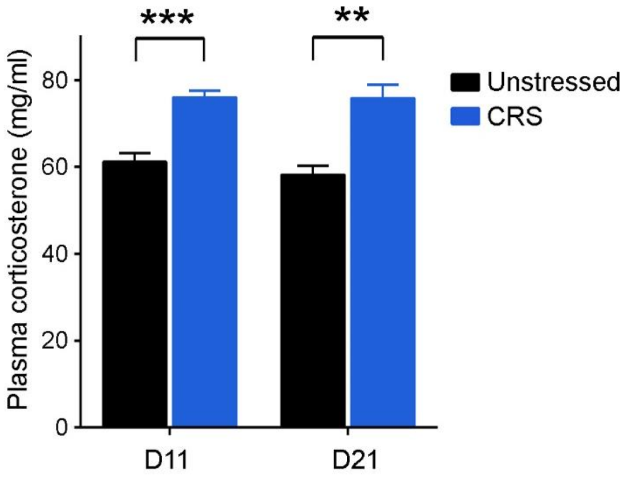

Supplementary Figure S2. Chronic restraint stress increases plasma corticosterone levels. Experimental mice were placed in 50-mL perforated conical centrifuge tubes for 7.5 h daily for 11 or 21 consecutive days. Plasma levels of corticosterone were measured on day (D) 11 and 21. Unstressed: mice allowed to move freely during the course of the experiment; CRS: chronic restraint stress-induced mice (n = 5 mice per group, **$p < 0.01$, ***$p < 0.001$, Student's t-test).

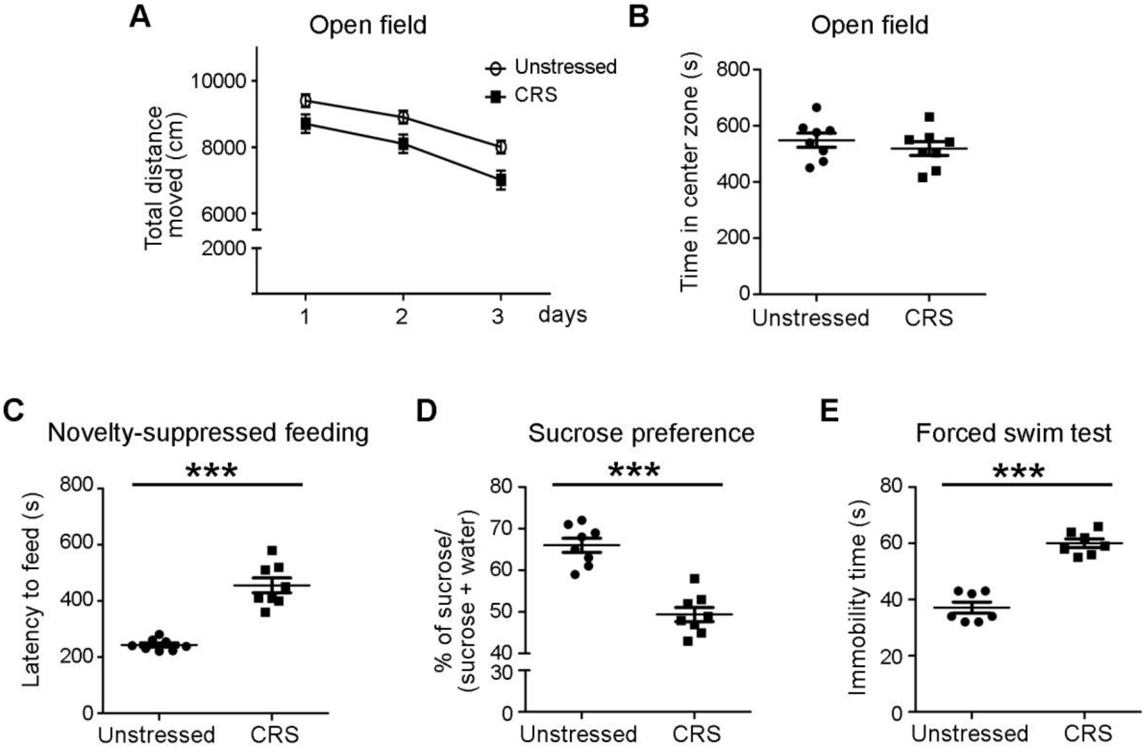

Supplementary Figure S3. Chronic restraint stress induces depression-like behaviors in mice. (A) Motor activity of mice subjected to chronic restraint stress (CRS) or freely moving mice (unstressed) during the 3-day open field test. (B) There was no difference between mice exposed to CRS and unstressed mice with regard to exploration of the center zone of a novel arena. (C) Mice subjected to CRS exhibited an increased latency to feed in the novelty-suppressed feeding test. (D) Mice subjected to CRS exhibited reduced sucrose consumption. (E) Mice subjected to CRS exhibited increased immobility time in the forced swim test. Unstressed: mice allowed to move freely during the course of the experiment; CRS: chronic restraint stress-induced mice (n = 8 mice per group, ***$p < 0.001$, Student's t-test).
